# Yeast Surface Display System: Strategies for Improvement and Biotechnological Applications

**DOI:** 10.3389/fbioe.2021.794742

**Published:** 2022-01-10

**Authors:** Karla V. Teymennet-Ramírez, Fernando Martínez-Morales, María R. Trejo-Hernández

**Affiliations:** Centro de Investigación en Biotecnología, Universidad Autónoma del Estado de Morelos, Cuernavaca, Mėxico

**Keywords:** cell surface display, yeast, anchor, microbial engineering, whole cell biocatalyst

## Abstract

Yeast surface display (YSD) is a “whole-cell” platform used for the heterologous expression of proteins immobilized on the yeast’s cell surface. YSD combines the advantages eukaryotic systems offer such as post-translational modifications, correct folding and glycosylation of proteins, with ease of cell culturing and genetic manipulation, and allows of protein immobilization and recovery. Additionally, proteins displayed on the surface of yeast cells may show enhanced stability against changes in temperature, pH, organic solvents, and proteases. This platform has been used to study protein-protein interactions, antibody design and protein engineering. Other applications for YSD include library screening, whole-proteome studies, bioremediation, vaccine and antibiotics development, production of biosensors, ethanol production and biocatalysis. YSD is a promising technology that is not yet optimized for biotechnological applications. This mini review is focused on recent strategies to improve the efficiency and selection of displayed proteins. YSD is presented as a cutting-edge technology for the vectorial expression of proteins and peptides. Finally, recent biotechnological applications are summarized. The different approaches described herein could allow for a better strategy cascade for increasing protein/peptide interaction and production.

**GRAPHICAL ABSTRACT:**
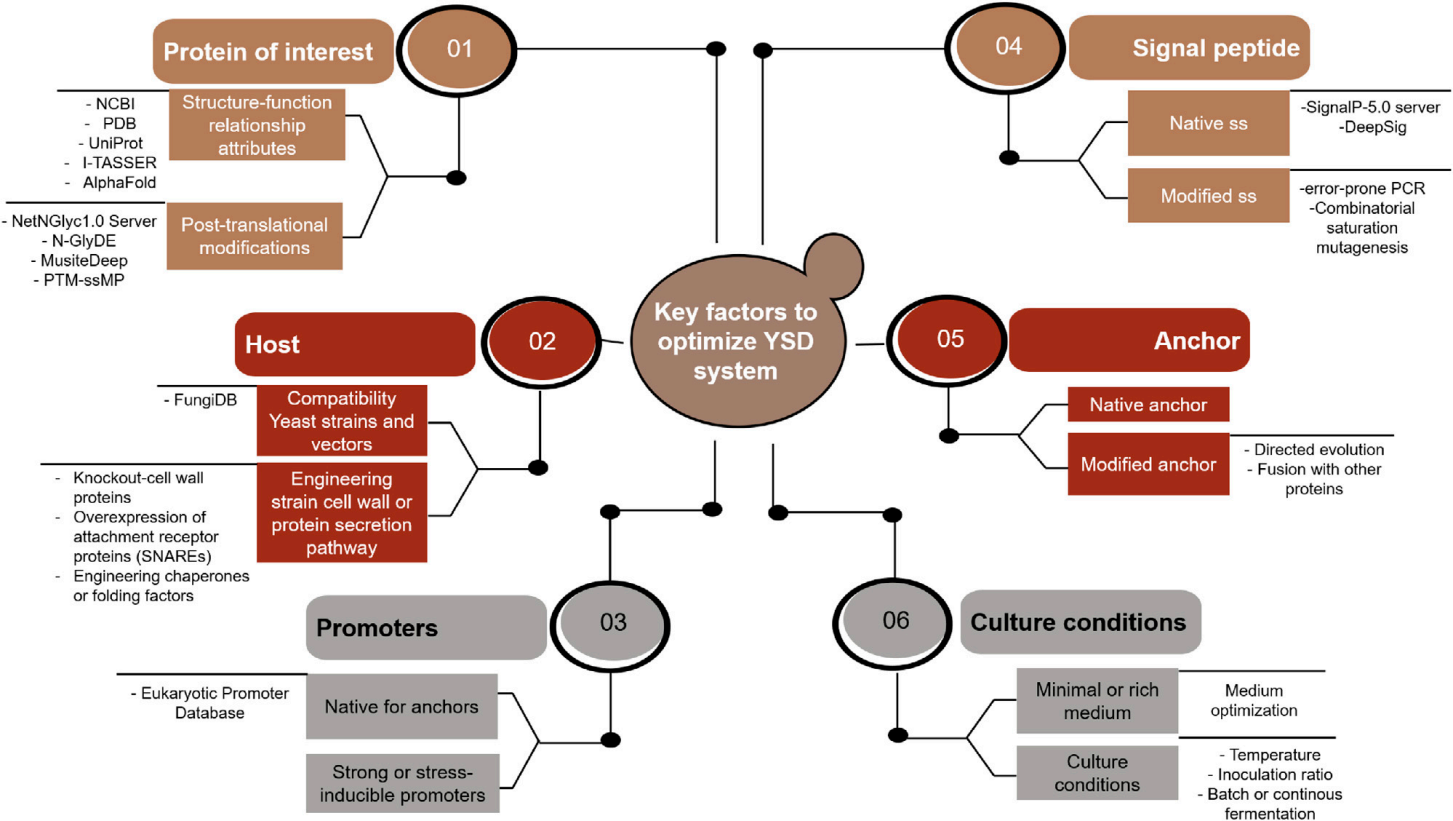
Different strategies and tools involved in the improvement of the YSD system performance. Abbreviatures found in the figure: NCBI (National Center for Biotechnology Information); PDB (Protein Data Bank); SS (signal sequence); CWPs (cell-wall proteins); SNARES (soluble N-ethylmaleimide-sensitive factor attachment receptor proteins); and CSM (combinatorial saturation mutagenesis).

## Overview of Yeast Surface Display

Cell-surface display allows the expression of target peptides or proteins on the cell surface of bacteria, yeast, insect, or mammalian cells through the connection of a protein of interest (POI) fused to an anchor protein. In yeast, typically, this comprises a cell wall protein (CWP) linked to glycosylphosphatidylinositol (GPI) (Tanaka and Kondo, 2015; Lim et al., 2017). This platform combines fine-tuned gene expression and protein immobilization, which simplifies the purification process and allows the reuse and recovery of biocatalysts (Yuzbasheva et al., 2011; Liu et al., 2014; Yang et al., 2019).

The yeast cell wall landscape consists of an internal layer, mainly composed of polysaccharides, and a 100–200 nm thick fibrillar outer layer framework of 50% mannoproteins, 30–45% β-1,3 glucans, 5–10% β-1,6 glucans and 1.5–6% chitin (Klis et al., 2006; Inokuma et al., 2014; Ananphongmanee et al., 2015). The cell surface offers a topological environment with unique properties not found in other cell compartments. The high content of polysaccharides allows multiple interactions with the proteins embedded in the cell wall that can have a positive impact on the properties of the POI although negative effects have also been reported depending on the yeast strain used for display (Crowe et al., 1988; Allison et al., 2000; Li et al., 2010).

Yeast surface display has been extensively used due to several advantages over other, similar systems, which includes: 1) various yeast strains (*Saccharomyces cerevisiae*, *Pichia pastoris* and *Yarrowia lipolytica*) have a “generally recognized as safe” (GRAS) status by the United States Food and Drug Administration (FDA), 2) yeast cells are able to perform eukaryotic post-translational modifications, 3) the ease of cell culture and genetic modification allows the proper folding and secretion of large and complex protein scaffolds, and 4) compatibility with flow cytometric analysis (Angelini et al., 2015; Lim et al., 2017; Raeeszadeh-Sarmazdeh et al., 2019).

When soluble proteins or peptides are linked to the cell surface, they gain biotechnological advantages not met by the soluble state, e.g. ease of recovery, control of the spatial protein orientation, concerted protein interactions with co-displayed proteins to mimic supramolecular complexes and easy cell sorting formats. Another important advantage of this system is the possibility of coupling yeast metabolism with the function of the protein on the yeast cell surface. This can be useful for the study of transport systems, signal transduction proteins, capsule influences and biofuel production (Wen et al., 2010).

It has been reported that biochemical and catalytic properties can be improved by immobilizing a protein on the yeast cell surface (Shiraga et al., 2005; Li et al., 2014; Moura et al., 2015). Since the first YSD system was developed by Boder and Wittrup (1997), this platform has been employed for the directed evolution of antibodies, peptides and proteins. Nevertheless, promising results in biotechnological applications have been achieved by engineering YSD in different ways that will be discussed herein.

## Strategies to Improve Yeast Surface Display

The directed evolution of proteins, antibodies and enzymes to increase their biochemical or catalytic properties has been discussed in excellent reviews (Traxlmayr and Obinger, 2012; Könning and Kolmar, 2018; Linciano et al., 2019). The strategies to improve the displayed protein expression and secretion by engineering the YSD directly are summarized in this review. Table 1 compares these strategies and their relevance on protein expression levels and/or activity.

**TABLE 1:** Strategies focused on key factors that affect the displayed protein production.

Strategy	Target protein	Yeast strain	Promoter	Signal sequence	Anchor	Main observations/conclusions	References
Promoters	β-glucosidase and endoglucanase II	*S. cerevisiae*	TDH3	Native signal sequence of *R. oryzae* gene	SAG1	Gene cassettes which contained SED1 promoter and anchor produced higher glucoamylase activity than SAG1+TDH3 promoter	Inokuma et al. (2014)
		BY4741	SED1		SED1		
	α-1,2- mannosidase	*Yarrowia lipolytica*	TEF1	Lip2 and Xpr2 prepro region, α-amylase SS rice α-amylase SS	GPI anchoring motifs	The highest efficiency was obtained with Lip2 prepro sequence	Moon et al. (2013)
	ZZ domain from *Staphylococcus aureus* protein A	*S. cerevisiae* BY4741	PGK1	α-factor	Flo1-derived anchor	Significant increase in protein display efficiency was obtained using the PGK1 promoter compared to GAL1	Katsurada et al. (2021)
	Pediocin PA-1	*S. cerevisiae*	GAPDH	SSS	α-agglutinin	Highest yield of cells expressing the pediocin (∼93%) was achieved by using the vector and grown cells in basic medium	Nguyen et al. (2020)
		W303					
Signal peptide optimization	Anti-hen egg-white lysozyme nanobody cAbLys3	*S. cerevisiae*	GAP	α-prepro leader	α-agglutinin	Highest fluorescence intensity was observed with 649-stalk anchor and α-prepro signal ss and GAP1 was stronger than pGAL1	Kajiwara et al. (2020)
		BY4741	GAL1		649-stalk		
	scFv antibody	*S. cerevisiae*	GAL10	α mating factor 1 leader peptide (MFα1pp)	-	Directed evolution on MFα1pp, obtaining 16-fold improvement over wild type	Rakestraw et al. (2009)
		BJ5464α					
Anchors	β-glucosidase and endoglucanase	*S. cerevisiae*	SED1	SED 1 SS	SED1	Specific BGL activity was ∼400U/g dry cells, while endoglucanase relative activity was improved 1.7-fold	Inokuma et al. (2021)
					SAG1		
	Xylose reductase	*S. cerevisiae*	GAL1		CCW12	Xylose reductase fused to N-terminal of Pir4 showed higher affinity for xylose than the construct with CCW12	Hossain et al. (2019)
			PHO5		Pir4		
	Luciferase	*S. cerevisiae* BY4741	Spi1 (regulated by stress) PGK1	Spi1 SS	Spi1	Spi1 promoter was not as strong as PGK1, but it can be induced by stress	Andreu and del Olmo, (2017)
Multi-enzyme assembly (Co-display)	EG, CBH, BGL	*P. pastoris*	AOX	-	SED + Im7 protein + CL7 protein	The assembly EG, CBH, BGL was functionally expressed, and 5.1 g/L ethanol was obtained	Dong et al. (2020)
		GS115					
	EG, CBHI, CBHII, BGL	*S. cerevisiae*	GAL1	-	Aga2 aScafs	Synergistic effects were observed when inter-enzyme distance in the multi-enzyme assembly is ∼130 nm	Smith et al. (2019)
		EBY100					
	β-Amylase and α-transglucosidase	*Y. lipolytica* CGMCC7326	-	-	Pir1	Functional expression of both enzymes and 75% of isomalto-oligosaccharides was obtained using the YSD	Liu et al. (2019)
Yeast cell wall modification	BGL and EG	*S. cerevisiae*	TEF1	SED1 SS	SED1	When CCW12 and CCW14 were co-knockout, 1.4-fold BGL activity was achieved	Inokuma et al. (2021)
		BY4741			SAG1		
	BGL and EG	*S. cerevisiae*	GAPDH	*R. oryzae glucoamylase* SS	Flo1	Highest activity for BGL and EG was achieved when mnn2 deletion strain was evaluated	Matsuoka et al. (2014)
		BY4741			α-agglutinin		
Secretory pathway modification	EG and BGL1	*S. cerevisiae*	TEF1	-	-	Over-expression of components related to vesicle trafficking (Sso1p, Snc2p, Sec1p) increased the BGL1 secretion	Tang et al. (2017)
		CEN.PK102-5B					
	Lipase B (CALB)	*P. pastoris*	AOX1	α-factor secretion signal	Screening of putative GPI-anchored proteins	13 GPI-modified cell wall proteins were confirmed in *P. pastoris,* Gcw61p being one of the best proteins for the lipase B	Zhang et al. (2013)
		GS115					
Novel configurations	GFP and human arginase I	*P. pastoris*	AOX1	-	SED + Im7 protein + CL7 protein	Functional expression of arginase I	Li et al. (2019)
		GS115					
	Exoglucanase	*S. cerevisiae*	TEF1	SUC2 SS	Aga1 without Aga2 subunit	Enzyme activity was improved 39% by fusing the POI directly on Aga1 with a flexible linker	Yang et al. (2019)
		CEN.PK102-5B					

EG: endoglucanase, BGL: β-glucosidase, GFP: green fluorescent protein, CBHI: reducing-end-cleaving cellobiohydrolase, CBHII: non-reducing-end-cleaving cellobiohydrolase.

### Genetic Strategies to Improve Yeast Surface Display

The regulatory and structural elements that control YSD can be organized in synthetic expression plasmids or integrated into the yeast genome. Usually, plasmid vectors are the first choice to assess the function of regulatory and structural DNA sequences. The genetic construct can be integrated into the chromosome to gain a more stable genetic background, generally not offered by episomal constructs.

#### Yeast Plasmids

Synthetic yeast plasmids are extrachromosomal genetic elements used for the controlled heterologous protein expression, designed to drive gene expression under the control of regulatory sequences, i.e., promoters, terminators, transcription factors, among others. Additionally, plasmid copy number can affect the level of gene expression (Redden et al., 2015). YSD plasmid protein expression depends on promoter strength (Inokuma et al., 2016). Both constitutive and inducible promoters have been used for displaying proteins. The most common promoters used are the galactose-promoter (GAL1/GAL10) for expression in *S. cerevisiae* (Schröter et al., 2018; Zhao et al., 2020b), GAP and AOX1 promoters for expression in *Pichia pastoris* (Yang et al., 2017; Li et al., 2019), and TEF1 and hp4d promoters for expression in *Y. lipolytica* (Yuzbasheva et al., 2011; Moon et al., 2013). Recently, GAPDH, GPD or stress-induced (SED1) promoters have been used for protein expression in *S. cerevisiae* (Inokuma et al., 2014; Zhang et al., 2019; Nguyen et al., 2020), proving to be appropriate alternatives to the galactose-induced promoter. One drawback is that the promoter strength may vary in different yeast genetic backgrounds (Inokuma et al., 2014; Andreu and del Olmo, 2017). The recent development of software, based on synthetic and omics approaches, predicts *in silico* and *in vivo* changes in the level of expression can give substantial information to rationally modify conditions to optimize the selection of a particular promoter (Fiore et al., 2015).

#### Signal Peptide Sequence

Generally, an anchor contains 2 main parts: 1) a signal peptide sequence (SS), involved in protein transport through the protein secretion pathway, and 2) an anchor to which the POI is fused (Tanaka and Kondo, 2015). Modification of SS has an important impact over the improvement in production levels of the displayed POI. Generally, native signal peptides are used, i.e., a-agglutinin, SED1p, Pir1p and Flo1p have been shown to give good protein expression levels using their own signal peptides (Khasa et al., 2011; Andreu and del Olmo, 2017). Likewise, SS from other genes have been evaluated, for example, the SS of *Rhizopus oryzae* glucoamylase or the SS of *Aspergillus niger* α-amylase (Moon et al., 2013; Inokuma et al., 2014). Recent studies on directed evolution of signal peptides have shown that changes in the hydrophobic core of the SS significantly impacts protein secretion (Mateljak et al., 2017; Barrero et al., 2018; Aza et al., 2021). It is worth mentioning that SS modifications can affect the protein secretion pathway, as discussed later.

#### Anchor Proteins

Selection of the anchor protein is crucial for display effectiveness and is dependent on the specific application and properties of the POI (Van der Vaart, 1997; Tanaka et al., 2012; Tanaka and Kondo, 2015; Yang et al., 2019). The most common anchor are the GPI-dependent CWPs (cell wall proteins), which provide a covalent bond between the target protein and the cell wall β-1,6 glucans. On the other hand, Pir-CWP contributes to the covalent linkage of fusion proteins both to cell wall β-1,3 glucans and to structural proteins via disulfide bonds (Duquesne et al., 2014). The Aga1-Aga2 anchor, initially developed by Boder and Wittrup (1997), has been used in the expression of several proteins (Blazic et al., 2013; Bertrand et al., 2016). Particularly in *P. pastoris*, SED1p and Pir anchors have shown the highest display efficiencies (Duquesne et al., 2014; Li et al., 2019; Dong et al., 2020).

**FIGURE 1:**
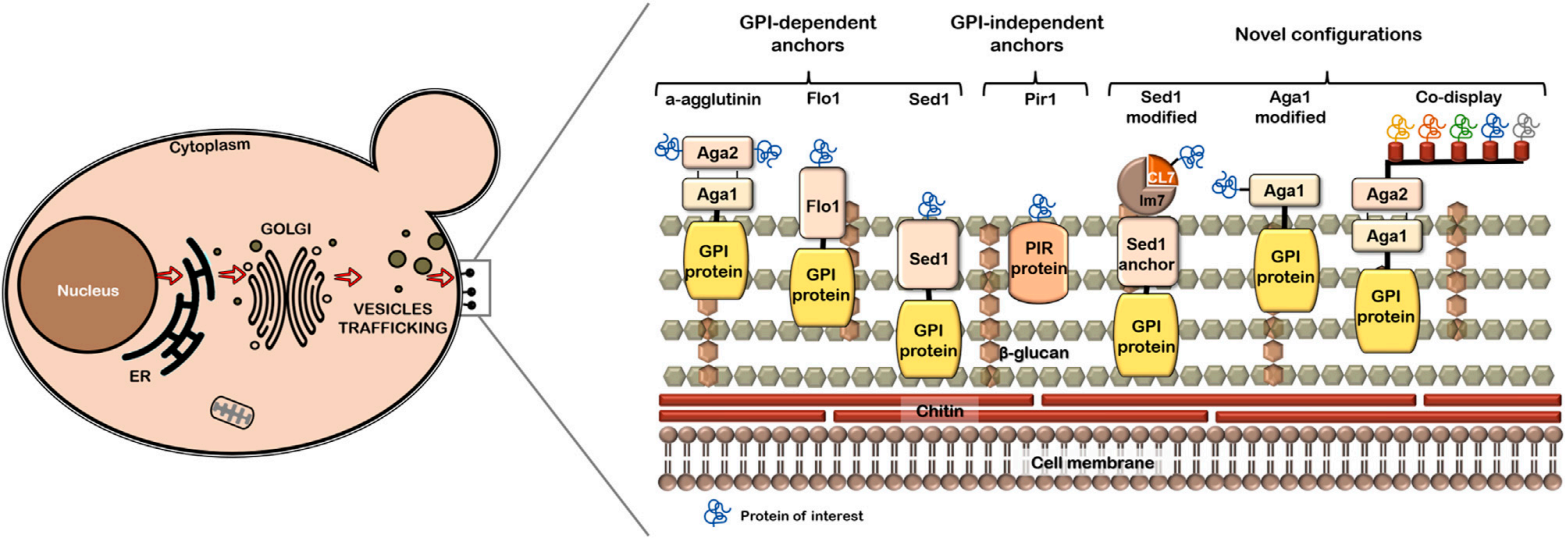
Scheme of cell wall proteins used as anchors in YSD system. Common anchors and the modified ones are shown in a representation of yeast cell wall.

Figure 1 depicts common and modified anchors mentioned in this review. Some anchors, such as Aga1-Aga2, allow the immobilization of proteins through their N-terminus or C-terminus. Optimal orientation of the POI has an impact on ligand binding affinity (Wang et al., 2005; Valldorf et al., 2021). Native or modified anchors have been successfully evaluated. A novel modified anchor (Li et al., 2019), which consists of the incorporation of the lm7 protein and CL7 protein between the SED1 anchor and the POI efficiently displays the green fluorescent protein (GFP) and a human arginase. Dong et al. (2020) also used the same assembly, SED1/Im7/CL7, to construct a minicellulosome assembly by endoglucanase, exoglucanase and β-glucosidase, which demonstrates the flexibility of this modified anchor for single or multiple enzyme display. Additionally, the search for novel GPI anchors has been performed in *P. pastoris* and *H. polymorpha* to find anchors with potential uses for YSD in other yeast strains (Zhang et al., 2013; Cheon et al., 2014).

#### Linkers or Spacers

The anchor length is another important factor to be considered, since the POI fused to short anchors may show steric hindrance of the active site, affecting ligand-receptor interactions (Sato et al., 2002). It is common to find small sequences between the anchor protein and the POI, called linkers or spacers. Linkers are *O*-glycosylated regions, which gives protection from protease degradation. The main function of linkers is the reduction of the effects of GPI fusion on the activity of the POI. A Ser/Thr-rich sequence and (G₄S)₃ linkers are the most widely used (Washida et al., 2001). It has been reported that the presence of linkers improves the display efficiencies and the activity of POI by preserving the conformation of the active site and the accessibility of substrates to the POI displayed at the outer yeast cell surface (Breinig and Schmitt, 2002; Sato et al., 2002; Liu et al., 2010; Yang et al., 2019). The anchor length enhancement and the addition of linkers between the anchor and the POI demonstrated a positive effect on accessibility of substrates to displayed proteins (Sato et al., 2002). Yang et al. (2019) reconstructed the a-agglutinin (Aga1-Aga2) by fusing the POI to the N-terminus of Aga1p and incorporating a flexible linker, containing 17 amino acids composed of Ser and Gly repeat sequence, between the POI and the anchor. This modification showed higher display efficiency and could be more suitable for larger proteins or protein complexes.

#### Multi-Enzyme Expression

A strategy widely evaluated is the co-display, which enhances the expression of the target protein or a complex of multiple proteins (Sun et al., 2012). Cellulases can be anchored to yeasts used in fermentations, allowing a formally two-step process to be carried out as a one-step process for a consolidated bioprocessing development (Wen et al., 2010). In a displayed multi-enzyme complex, the distance between the enzymes and the number and nature of cohesion domains must be considered (Tsai et al., 2013). Enzyme-substrate interactions can be negatively affected if distance is not optimum, the opposite effect is detected when a favorable distance is established. Smith et al. (2019) highlighted that the enzyme density is a pivotal parameter to enhance cellulose hydrolytic performance when a multi-enzyme assembly is designed. Tsai et al. (2013) observed a 2-fold increase in ethanol production when cells displayed a tetravalent cellulosome instead of a divalent cellulosome. Novel protein complexes not found in nature can be designed on the surface of yeast or other cell systems by exploiting the extensive proteomic databases available today, e.g., Proteomics DB (https://www.proteomicsdb.org) (Tunyasuvunakool et al., 2021).

### Yeast Strain Selection for Yeast Surface Display

The genomic and metabolic background of the applied yeast strain is an important trait that influences YSD of native or heterologous proteins. Synthetic biology approaches combined with genomic techniques (e.g., CRISPR/Cas system) have allowed the development of yeast strains with fine-tuned heterologous protein expression. Novel yeast strains for YSD are designed by modifying their native features, such as their cell wall composition or their protein secretion pathway. The latter includes the engineering of vesicle trafficking and the development of a platform that secrete simultaneously soluble protein and surface-displayed protein to ease their characterization (Zhang et al., 2013; Cruz-Terán et al., 2017; Tang et al., 2017). *S. cerevisiae* strains, mainly EBY100 (ATCC MYA-4941) and BY4741 (Brachmann et al., 1998) have been successfully used as hosts for YSD, MATa and the ura3 gene disruption (used as an auxotrophic marker) being the main features of these strains. In addition, to obtain an optimized system other yeast strains have been evaluated, e.g., *S. cerevisiae* (AWY100, AWY101, AWY102), *Pichia pastoris, Yarrowia lipolytica, Hansenula polymorpha* and *Saccharomyces boulardii* (Cheon et al., 2014; Dong et al., 2020; Wang et al., 2020; Patent CN103031329B, 2014; Wentz and Shusta, 2007).

Some authors propose modifications of cell wall protein composition to obtain a suitable environment for display. However, one genetic defect can cause pleiotropic changes in the cell wall structure-function relationship (Matsuoka et al., 2014). Inokuma et al. (2021) increased the cell wall thickness of *S. cerevisiae* by performing the knockout of proteins CCW12 and CCW14, which allowed an increased amount of displayed β-glucosidase as compared to the parental strain.

For the secretion of heterologous proteins is the protein secretory pathway, which includes protein translocation, protein folding, post-translational modifications, protein sorting and trafficking. The secretory pathway can be influenced solely by the nature of SS (Tang et al., 2015). Additionally, the number of proteins that reach cell surface can be controlled by changes in the number of molecules that travel through the secretion pathway. Foreign proteins can be diverted to a vacuolar compartment for destruction due to misfolding (Fitzgerald and Glick, 2014). Efforts related to engineering chaperones, folding factors and translocation components have been performed exhibiting improvements in the secretion of heterologous proteins (Tang et al., 2015; Duan et al., 2019). Tang et al. (2017) improved the surface display efficiency of cellulases by over-expressing the components involved in vesicle trafficking, such as Snc2p, Exo70p and Sso1p. On the other hand, a system based on inefficient ribosomal skipping allows the simultaneous expression of soluble and cell surface displayed proteins to simplify the screening of combinatorial protein libraries and protein characterization (Cruz-Terán et al., 2017).

### Strategies to Improve the Screening of Displayed Proteins

As previously mentioned, improvement in protein display efficiency is crucial for the implementation of YSD. However, a straightforward detection methodology for displayed proteins is also required for a good performance of combinatorial library screening. For example, GFP has shown high performance of yeast display library high-throughput screening of glucose oxidase (Kovačević et al., 2019). Uchański et al. (2019) developed a YSD platform for the screening of nanobody libraries. Their research group fused a nanobody to the N-terminal of Aga2p to avoid steric hindrance and nanobody detection was performed by using a fluorophore which was attached to an orthogonal acyl carrier protein tag by a one-step reaction catalyzed by a Sfp synthase. In addition, it has been reported that the visualization of endoglucanase displayed on the yeast surface can be performed by using atomic force microscopy (Takenaka et al., 2017).

### Yeast Surface Display Combined With Microbial Engineering Approaches

Protein properties such as stability and activity can be improved by displaying them at yeast cell surface (Shiraga et al., 2005; Li et al., 2014). It is possible to improve the biochemical and catalytic properties of the POI by combining protein engineering with YSD (Antipov et al., 2008; Chen et al., 2011) or to increase the production of value-added chemicals by metabolic engineering (Takayama et al., 2018). Bacon et al. (2019) fused YSD with nanotechnology for the screening of combinatorial libraries. The group co-expressed the target protein with a protein showing affinity to iron oxide for separation of cells by magnetism. A combination of YSD with SELEX technology has aided in the characterization of endonucleases (Jacoby et al., 2017).

### Trends in the Application of Yeast Surface Display

YSD can be used in a wide range of applications, such as the engineering proteins or peptides (Smith et al., 2015; Deweid et al., 2018; Raeeszadeh-Sarmazdeh et al., 2019), for whole-cell biocatalysis (Zheng et al., 2019; Wang et al., 2020), production of vaccines (Lei et al., 2016), antibody and nanobody production (McMahon et al., 2018; Sun et al., 2019), biofuel production (Yang et al., 2017; Dong et al., 2020), biofuel cells (Fan et al., 2020) and whole-proteome studies (Bidlingmaier and Liu, 2007; Procko, 2020). A description of some recent advances in YSD biotechnology applications is presented in this section.

Recent reviews on antibody engineering include novel strategies using approaches such as yeast mating and yeast endoplasmic reticulum sequestration screening (YESS) (Könning and Kolmar, 2018; Valldorf et al., 2021). The following applications focus mainly on enzyme biotechnology.

Recent advances in human health care include a vaccine against candidiasis (Shibasaki et al., 2013; Ueda, 2016) and a platform to detect SARS-CoV-2 (Maneira et al., 2021). Animal vaccines have been developed which include vaccines against *Toxoplasma gondii* (Wang et al., 2018), a vaccine against hemorrhagic disease of grass carp (Luo et al., 2015) and an anti-tick vaccine (Trentelman et al., 2021). Other relevant applications are the development of antibiotics (Chun et al., 2020), biosensors to detect blood biochemical parameters (Zhao et al., 2020b) and the expression of hydrophobins (Andreu et al., 2021).

In the food industry YSD can be used to immobilize enzymes that synthesize relevant compounds to produce sweeteners such as isomaltulose and fructooligosaccharides (Zhang et al., 2016; Zheng et al., 2019) and beneficial fats, e.g., omega 3-fatty acids (Singh et al., 2020). Additionally, whole-cell biocatalysts have been used to improve the sensory properties of beverages which include beer and wine (Cejnar et al., 2017; Zhang et al., 2019). In agri-food applications YSD can be used to develop biocontrol agents, e.g., expressing flagellin to increase the resistance of tomatoes towards *Botrytis cinerea* infection (Zhao et al., 2020a).

Related to bioethanol production, YSD can exploit the metabolic ability of yeast to ferment sugars to ethanol and the activity of hydrolytic enzymes immobilized on the yeast surface to achieve the simultaneous cellulose saccharification and ethanol fermentation for a consolidated bioprocessing (Fan et al., 2012; Tsai et al., 2013; Ishii et al., 2016; Chen, 2017; Khatun et al., 2017; Yang et al., 2017; Bamba et al., 2018; Tang et al., 2018; Anandharaj et al., 2020; Dong et al., 2020). Yuzbasheva et al. (2015) performed biodiesel production using the lipase Lip2 displayed by *Y. lipolytica*. The aforementioned examples couple the cell-surface exposure of hydrolytic enzymes with the yeast robustness to efficiently ferment sugars. Also, the yeast GRAS attributes allow a wide use in this and other fields.

## Conclusions

Immobilized enzymes by YSD present a wide range of applications, converting YSD to a powerful alternative to conventional immobilization. It allows the advancement of engineered microorganisms, with special functions not found in nature, for biotechnological applications. Since developed the first YSD, different strategies have been followed to improve the production and detection of the POI in this platform. Particularly, it was demonstrated that anchors have a decisive impact on YSD of fully functional proteins. Additionally, the compatibility of the YSD system with metabolic or protein engineering offers vast opportunities for the introduction of new platforms fed by omics databases and predictive software coupled to *in vivo* or *in vitro* systems. It is still a long way to obtaining an optimal YSD platform, but the existing strategies are the foundations for the development of new bioengineering strategies.
